# Effect of Electrolyte Concentration on Cell Sensing by Measuring Ionic Current Waveform through Micropores

**DOI:** 10.3390/bios11030078

**Published:** 2021-03-12

**Authors:** Kazumichi Yokota, Muneaki Hashimoto, Kazuaki Kajimoto, Masato Tanaka, Sanae Murayama, Makusu Tsutsui, Yoshihiro Nakajima, Masateru Taniguchi, Masatoshi Kataoka

**Affiliations:** 1 National Institute of Advanced Industrial Science and Technology, Takamatsu, Kagawa 761-0395, Japan; kazumichi-yokota@aist.go.jp (K.Y.); muneaki-hashimoto@aist.go.jp (M.H.); k-kajimoto@aist.go.jp (K.K.); mst-tanaka@aist.go.jp (M.T.); y-nakajima@aist.go.jp (Y.N.); 2 The Institute of Scientific and Industrial Research, Osaka University, 8-1 Mihogaoka, Ibaraki, Osaka 567-0047, Japan; murayama@sanken.osaka-u.ac.jp (S.M.); tsutsui@sanken.osaka-u.ac.jp (M.T.); taniguti@sanken.osaka-u.ac.jp (M.T.)

**Keywords:** resistive pulse method, cell discrimination, cancer cell, leukocyte, micropore

## Abstract

Immunostaining has been widely used in cancer prognosis for the quantitative detection of cancer cells present in the bloodstream. However, conventional detection methods based on the target membrane protein expression exhibit the risk of missing cancer cells owing to variable protein expressions. In this study, the resistive pulse method (RPM) was employed to discriminate between cultured cancer cells (NCI-H1650) and T lymphoblastoid leukemia cells (CCRF-CEM) by measuring the ionic current response of cells flowing through a micro-space. The height and shape of a pulse signal were used for the simultaneous measurement of size, deformability, and surface charge of individual cells. An accurate discrimination of cancer cells could not be obtained using 1.0 × phosphate-buffered saline (PBS) as an electrolyte solution to compare the size measurements by a microscopic observation. However, an accurate discrimination of cancer cells with a discrimination error rate of 4.5 ± 0.5% was achieved using 0.5 × PBS containing 2.77% glucose as the electrolyte solution. The potential application of RPM for the accurate discrimination of cancer cells from leukocytes was demonstrated through the measurement of the individual cell size, deformability, and surface charge in a solution with a low electrolyte concentration.

## 1. Introduction

The resistive pulse method (RPM), which is used to evaluate the transient ionic current blockade associated with the translocation of an individual nano- to micro-sized particle passing through a pore, can probe small objects as pulse-like electrical signals. These objects can be discriminated at a single-particle resolution because the measured ionic current blockade signals possess information regarding the physical properties of these particles, such as size [1], shape [2,3,4], surface charge [1,5], and deformability [6,7]. This technology is utilized for the discrimination of single-bioparticles of various sizes, ranging from blood cells to polynucleotides, without implementing immunostaining [2,8,9,10]. Micropore devices with micro-sized pores are mainly used for counting the number of cells and measuring their size [11]. They are used in hematological diagnosis to measure the number of blood cells, which is indicated by a Coulter counter.

The detection of rare cells in blood such as circulating tumor cells (CTCs), as well as counting the number of cells, is useful for clinical diagnosis [12]. CTCs are recovered from blood by centrifugation into leukocyte fraction, which can predict metastasis and determine drug efficacy. Although the identification of cancer cells from leukocytes at the single-cell level for the quantitative detection of CTCs focusing on biological properties with immunostaining has been put into practical use as a CellSearch system, cancer cells may be overlooked when the antigen expression is low [13,14]. Meanwhile, several methods for distinguishing leukocytes from cancer cells focusing on physical characteristics such as cell size, which does not depend on immunostaining, have been reported; however, an accurate discrimination is difficult [15]. Therefore, by changing the measurement conditions (such as the electrolyte concentration) when taking measurements using the micropore device, the accuracy of cell discrimination was improved owing to the difference in cell deformability and surface charge in addition to the difference in size. In this study, we demonstrate the accuracy of the RPM in distinguishing cancer cells from leukocytes.

## 2. Materials and Methods

### 2.1. Cell Cultures and Sample Preparation for Measuring Ionic Current

Human bronchioalveolar carcinoma cells (NCI-H1650) were cultured in Rosewell Park Memorial Institute media 1640 (Nacalai Tesque, Kyoto, Japan) containing 10% fetal bovine serum (FBS), 100 U/mL penicillin–streptomycin (GIBCO, Thermo Fisher Scientific Inc., Waltham, MA, USA), and 250 ng/mL Fungizone (GIBCO), and were subsequently harvested by centrifugation in trypsin. Human T lymphoblastoid leukemia cells (CCRF-CEM) were also cultured in a similar medium and harvested by centrifugation. Human gastric signet ring cell adenoma cancer cells (KATO-III) as other cancer cells were cultured in Iscove’s modified Dulbeco’s medium with L-glutamine and 4-(2-hydroxyethyl)-1-piperazineethanesulfonic acid (Nacalai Tesque) containing 20% FBS, 100 U/mL penicillin–streptomycin, and 250 ng/mL Fungizone, and were subsequently harvested by centrifugation in trypsin. To prepare the cell samples for ionic current measurements, we prepared cell suspensions of 1 × 10^7^ cells/mL in 1.0 × phosphate-buffered saline (PBS) and 0.5 × PBS containing 2.77% (*w*/*v*) glucose (Nacalai Tesque) (0.5 × PBS). Glucose was used to maintain the isotonic condition.

### 2.2. Fabrication of Microfluidic Cell Sensing Chips

**Figure 1:**
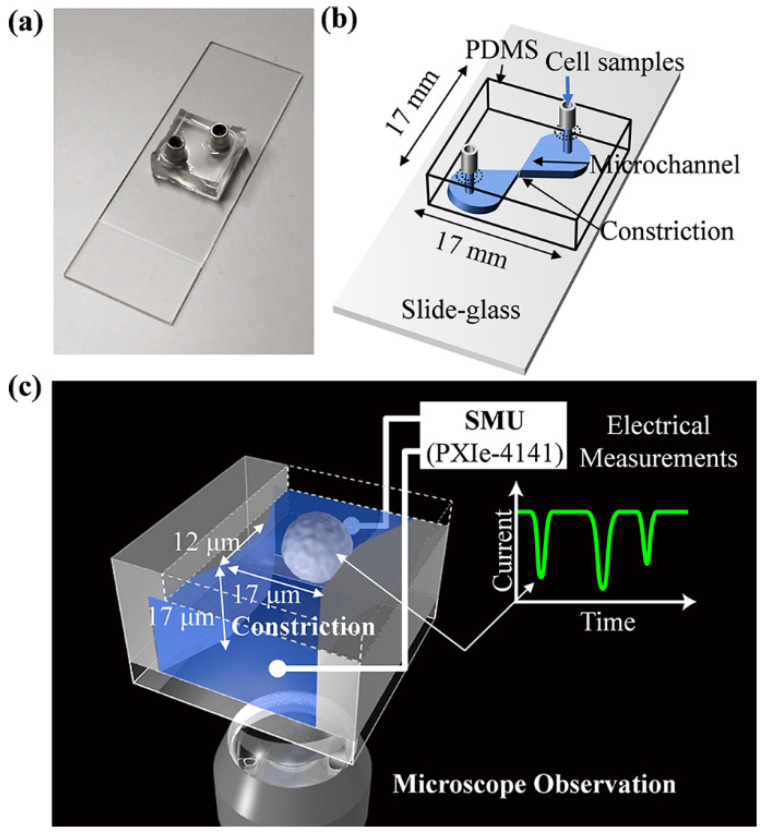
Microfluidic cell sensing chip. (**a**) Picture of microfluidic cell sensing chip made of polydimethyl siloxane (PDMS) fabricated on a slide glass. (**b**) Design of the mold used for the SU-8 microchannel. One constriction operating as a narrow flow path was formed. The microchannel was formed at the bottom of a replicated PDMS and sealed using a slide glass. The cell samples in 1.0 × phosphate-buffered saline (PBS) or 0.5 × PBS were injected from the inlet through a hole connecting the microchannel until it was full. For each analysis, 7 µL of electrolyte solution was added to the inlet reservoir to apply a pressure of 10 Pa. (**c**) Schematic illustration of ionic current blockade by resistive pulse method (RPM). Each constriction was 17 µm in width, 12 µm in length, and 17 µm in height. The translocation of cells through a constriction generated a pulse-like ionic current blockade, and electrical signals were then measured as a source measurement unit (SMU). The translocation of cells can be simultaneously observed using inverse optical microscopy.

The molds of the microchannels (17 μm in height) with constrictions (17 μm in width and 12 μm in length at the narrowest region) were formed with SU-8 photoresist (KAYAKU Advanced Materials, Inc. Tokyo, Japan) on a SiO_2_/Si wafer (Electronics and Materials Co. Ltd., Hyogo, Japan) using a conventional photolithography method [16]. The constriction sizes were chosen to have the smallest value that prevents the cells from getting stuck in the constriction (data not shown). The base and curing agents were mixed at a ratio of 10:1, and then polydimethyl siloxane (PDMS; SILPOT 184, Dow Corning Toray Co., Ltd., Tokyo, Japan) was poured on top of the mold, which was surrounded by 17 mm × 17 mm frames, and was cured at 100 °C for 35 min to fabricate a PDMS replica block. A block of PDMS replica was peeled from the mold, and 2 through-holes connecting the microchannel were punched out as the inlet and outlet for the samples. Each hole had a stainless-steel reservoir (Wurth Elektronik, Waldenburg, Germany) with an inner diameter of 3.0 mm. The fabricated block was bonded on a slide glass plate by activating both sides of the adhering surface through oxygen plasma exposure for 15 s under a 4 Pa pressure at 100 W (SC-708, Sanyu Electron Co., Ltd., Tokyo, Japan). The hydrophilizing agent poly(2-methacryloyloxylethyl phosphorylcholine (MPC)-*co*-2-ethylhexyl methacrylate (EHMA)-*co*-2-(*N*,*N*-dimethylamino)ethyl methacrylate (DAEMA)-*co*-poly(ethylene glycol) methacrylate (PEGMA) [17,18], named poly-(MPC-*co*-EHMA-*co*-DAEMA-*co*-PEGMA), was first injected through the inlet and then washed using water. Schematic illustrations of the microfluidic cell sensing chip are shown in Figure 1.

The microchannel in the fabricated chip possessed one constriction that was 17 μm in width, 12 μm in length, and 17 μm in height at the narrowest region for measuring the transient ionic current blockade by RPM (Figure 1b,c). An inverse optical microscope was installed at the constriction to observe the translocation of cells through the channel.

### 2.3. Cell Size Measurement by Using Optical Microscope

**Figure 2:**
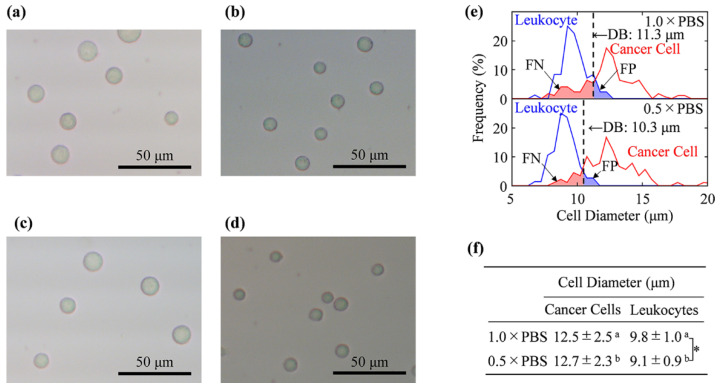
Cell size analysis by light microscopy. The microscope images of (**a**) cancer cells in 1.0 × PBS, (**b**) leukocytes in 1.0 × PBS, (**c**) cancer cells in 0.5 × PBS, and (**d**) leukocytes in 0.5 × PBS. (**e**) Histograms of the size distribution obtained for each cell evaluated using the microscopic images. (**f**) The average values and standard deviation for the cell diameters. The size of leukocytes in 0.5 × PBS is significantly smaller than that in 1.0 × PBS. A comparison between the two groups was performed using an unpaired *t*-test. The statistical significance is marked with * and a, b (*p* < 0.01).

To determine the size of cancer cells and leukocytes, we examined 100 cells in each cell suspension, namely, 1.0 × PBS and 0.5 × PBS containing 2.77% glucose, using an optical microscope (DIML II, Leica Camera AG, Wetzlar, Germany) with a 20 × objective lens. Approximately 20 μL of each cell sample was seeded into a 96-well plate (Nunc MicroWell 96-Well Microplates, Thermo Fisher Scientific Inc.) to settle at the bottom of the surface. The diameter of each cell was then measured immediately (Figure 2a–d); a histogram with a width of 0.5 μm was generated to evaluate the cell size distribution of each cell (Figure 2e,f).

### 2.4. Determination of Decision Boundary and Discrimination Error

**Figure 3:**
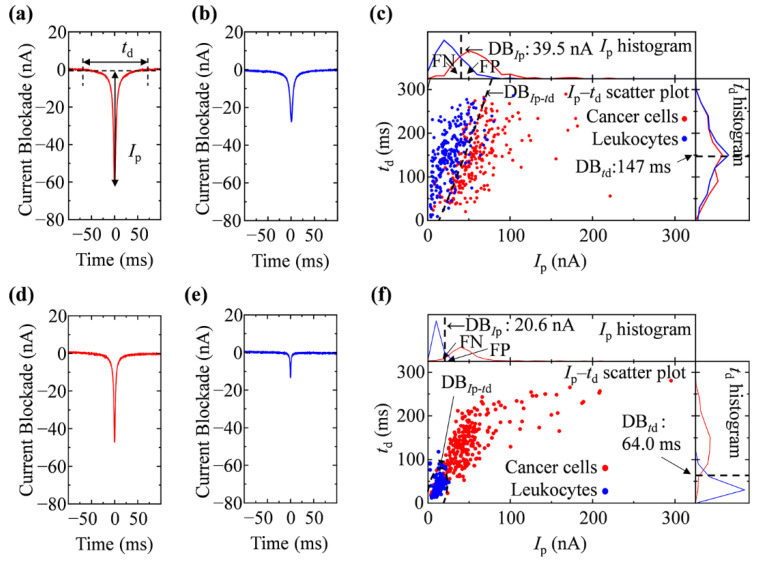
Electric current measurement by RPM. A typical waveform of the ionic current blockade by RPM was observed for (**a**) a cancer cell and (**b**) leukocytes in 1.0 × PBS. (**c**) The histogram of *I*_p_ and *t*_d_, scatter plot of *I*_p_–*t*_d_, and the decision boundary for these cells. A typical waveform of the ionic current blockade by RPM was observed for (**d**) a cancer cell and (**e**) leukocytes in 0.5 × PBS. (**f**) The histogram of *I*_p_ and *t*_d_, scatter plot of *I*_p_–*t*_d_, and the DBs for these cells.

A quadratic discrimination analysis (QDA) with the nonlinear separation boundary (Figure 2e and Figure 3c,f) is able to discriminate the different classes more accurately than the linear separation boundary [19]. QDA is a probabilistic parametric classification technique. It separates the class region by quadratic boundaries and assumes that each class has a multivariate normal distribution, while the dispersion is different in the classes. The decision boundary (DB) by QDA for cell discrimination is defined by a contour line/curve providing an equal probability of the *I*_p_, *t*_d_, and *I*_p_–*t*_d_ distribution for cancer cells and leukocytes [20,21]. The probability is expressed as follows:

(1) $$P\left(y_{i}\right)=\frac{1}{\sqrt{\left|2\pi \Sigma_{k}\right|}}\exp\left(-\frac{1}{2}{\left(y_{i}-{\langle y\rangle }_{k}\right)}^{T}\Sigma_{k}^{-1}\left(y_{i}-{\langle y\rangle }_{k}\right)\right)$$

where *Σ_k_* and <*y*>*_k_* are the variance–covariance matrix and the mean value of observations *y* for parameters of the *k*th dimension, respectively. The discrimination error was calculated by cross-validation as 100 × (FN + FP)/(TP + FN + FP + TN) [22]. Here, TP, FN, FP, and TN are the number of signals for true positive, false negative, false positive, and true negative cancer cells and white blood cells, respectively. The confidence interval (CI) for the discrimination error rate was evaluated using the Clopper–Pearson method [23].

### 2.5. Ionic Current Measurements

Before performing the measurements, the microchannels of the fabricated device were filled with 1.0 × PBS or 0.5 × PBS as the electrolyte solution. Then, 10 μL of cell samples corresponding to each electrolyte solution were injected through the inlet. For cell mixture measurement, each 5 μL of cancer cell and leukocyte samples were mixed and injected. For each analysis, 7 μL of the electrolyte solution was added to the inlet reservoir to apply a pressure of 10 Pa as a driving force for the cells to translocate into the microchamber from the inlet to the outlet. A pair of platinum (φ = 0.8 mm and 99.98 % purity, The Nilaco Corporation, Tokyo, Japan) electrodes coated with Ag/AgCl ink (ALS Co., Ltd., Tokyo, Japan) was then inserted through both the holes, each corresponding to the inlet and outlet. A bias voltage *V*_b_ was applied to the electrode at the outlet, while the inlet electrode was grounded. The ionic current was measured using a source measurement unit (SMU, NI PXIe-4141, National Instruments, Austin, TX, USA) at a current range of 10 μA with the LabVIEW (LabVIEW 2017, National Instruments) program. The time trace of the ionic current was evaluated via the constrictions and was recorded at a sampling rate of 50 kHz, which corresponds to a 20 μs resolution time. Typical wave forms of the ionic current blockade for cancer cells and leukocytes are shown in Figure 3a,b,d,e; the duration time of a current blockade signal was >≈10 ms and the employed sampling rate was sufficient to analyze each signal of the current blockade. Typical histograms are also shown in Figure 3c,f.

### 2.6. Resistive Pulse Analyses and Cell Discrimination

Resistive pulse signals that appeared on the time trace of the ionic current were searched by monitoring the current displacement, which was larger than the threshold by 3 times the standard deviation (SD). To reduce the current noise in the pulse search process, we averaged the data for 10 nearest neighboring points, as reported by Smeets et al. [24]. At the time point of pulse detection, the waveform of the pulses of the original 50 kHz data was extracted, and the baseline current level was offset to zero. The peak values of the current blockade (*I*_p_) and the duration time of the current blockades (*t*_d_) were evaluated on the extracted waveform (Figure 3a,b). These data processes were conducted using the LabVIEW program.

### 2.7. Zeta Potential Measurements

In RPM, *t*_d_ can be utilized to elucidate the surface charge of the sensed particles [1,5]. The surface charge of cells can be estimated by examining the zeta potential [25,26]. A zeta potential analyzer (ELSZ-2000Z Otsuka Electronics Co., Ltd. Osaka, Japan) was used for the measurements in this study. A glass flow cell was filled with 1.0 mL of cell sample at a concentration of 2 × 10^8^ cells/mL in 1.0 × PBS and 0.5 × PBS. While applying an electric field of ≈15 V/cm on average, we evaluated the electrical mobility from the doppler shift on the scattering light of the laser, and the zeta potential *ζ* was obtained by fitting the electrophoretic velocity of the cells flowing inside the measurement glass cell on the basis of the Smoluchowski equation [27].

## 3. Results and Discussion

### 3.1. Cell Size Examination by Light Microscopy

We employed cancer cells (NCI-H1650) that are relatively smaller compared to other cancer cells and overlap with leukocytes (CCRF-CEM) [15,28,29]. To examine the size of the cancer cells and leukocytes, we used light microscopy for the cell samples in 1.0 × PBS or 0.5 × PBS. Light microscopic images of these cells are shown in Figure 2a–d, and the histograms of the size distribution for these cells are shown in Figure 2e. Although the cancer cells in 1.0 × PBS were statistically discriminated from the leukocytes owing to their larger cell size (the average value and the SD for diameters of cancer cells were 12.5 ± 2.5 μm and those for leukocytes were 9.8 ± 1.0 μm, *p* < 0.01), an overlap in the cell size was also observed in the histogram (Figure 2e,f). The decision boundary (DB) for the cell discrimination in terms of cell size was evaluated at 11.3 μm, with a discrimination error of 17.5 % (Figure 2e, Table 1). 

This result was similar to that of CTC isolation, which relies on the cell size exclusion loss from 20 to 50 % of CTCs [15]. The DB and the discrimination error rate in 0.5 × PBS were 10.3 μm and 8.5%, respectively (Figure 2e, Table 1). The summarized mean value of each cell’s diameter is shown in Figure 2f. The mechanism for the significant decrease in the size of the leukocytes when a solution with a low electrolyte concentration is used is unknown; despite maintaining the isotonicity condition by adding glucose, the size of the leukocytes in 0.5 × PBS was approximately 7% smaller than that in 1.0 × PBS. When mannitol or sorbitol was used instead of glucose in 0.5 × PBS, we observed similar cell size decreases (data not shown); leukocyte cell size decrease was thought to be due to low salt stress. Therefore, the improvement in accuracy for cell discrimination in the solution with a low electrolyte concentration must have been due to the decrease in the size of leukocytes. 

### 3.2. Ionic Current Measurement of Cells

A biased voltage V_b_ = 1.0 V was applied between the inlet and outlet in 1.0 × PBS, and the baseline at a current level of approximately 2 μA was measured. The electrical conductivity (σ) of 1.0 × PBS was determined to be 1.41 ± 0.04 S/m. The electric field generated by the bias voltage yields electrophoretic forces to drive the cells and translocate them through the constrictions. As illustrated in Figure 1c, the cells were restricted by a constriction, causing them to pass one by one through the narrow space, which yielded a one-to-one correspondence of a pulse-like signal between the ionic current blockade and a translocation event of a cell through the constriction. Typical waveforms of the current blockades generated by the cancer cells and leukocytes are shown in Figure 3a,b, respectively. The size and deformability of the sensed particles were estimated from the peak height of the current blockade (*I*_p_) generated by the RPM [1,6,7]. As shown in Table 2, the mean values of *I*_p_ were significantly different (*p* < 0.01) for cancer cells and leukocytes (the mean value and SDs were 58.7 ± 1.9 nA and 26.3 ± 1.6 nA, respectively). 

Although the difference in *I*_p_ between the cancer cells and leukocytes were observed to be larger than that indicated by the results of the cell diameter examination by microscopy, the discrimination error rate based on the detection boundary for *I*_p_ (DB*_I_*_p_) was 27.3 ± 3.5% (Figure 3c, Table 1). Improvement in the accuracy of cell discrimination by *I*_p_ could not be observed compared to the microscopic observation. This may have been due to the relatively large variation in the distribution of *I*_p_ (Figure 3c). In RPM, the duration time for the current blockade (*t*_d_) can be utilized to elucidate the surface charge of the sensed particles [1,5]. The zeta potential was examined for the estimation of the surface charge of cells. The evaluated zeta potential for cancer cells and leukocytes in 1.0 × PBS were −10.1 ± 0.7 mV and −12.0 ± 0.6 mV (*p* = 0.048), respectively. As the differences in the zeta potential for cancer cells and leukocytes were confirmed, we further added *t*_d_ as the parameter for discrimination. The distribution of *t*_d_ is also shown in the *t*_d_ histogram in Figure 3c; the mean value and standard deviation of *t*_d_ for the cancer cells and leukocytes were 145.2 ± 1.9 ms and 150.0 ± 1.4 ms, respectively (Table 2). The discrimination error rate in terms of the detection boundary for *t*_d_ (DB*_t_*_d_) was calculated to be 45.1 ± 0.3%, and the accurate discrimination could not be estimated by *t*_d_ only (Table 1). The *I*_p_–*t*_d_ scatter plot was constructed using the measured waveforms for the individual cells, as shown in Figure 3c. By performing discriminant analysis on the *I*_p_–*t*_d_ plane, in which the decision boundary was indicated by the broken line as DB*_I_*_p_–*_t_*_d_, we obtained a discrimination error rate of 18.7 ± 4.0% (Table 1). The accuracy of discrimination by *I*_p_–*t*_d_ showed a significant improvement compared to *I*_p_ and *t*_d_ (*p* < 0.01); however, no improvement was observed compared to the microscopic examination.

In 0.5 × PBS (σ = 0.815 ± 0.005 S/m), we could apply a bias voltage V_b_ = 2.0 V to the baseline at a current level of approximately 2 μA, which is comparable to 1.0 × PBS. A typical waveform of the ionic current blockade for cancer cells and leukocytes in 0.5 × PBS is shown in Figure 3d,e; the *I*_p_ and *t*_d_ histograms and *I*_p_–*t*_d_ scatter plots are shown in Figure 3f. As shown in Table 2, the mean values of *I*_p_ were significantly different (*p* < 0.01) for cancer cells and leukocytes (the mean value and SDs were 50.8 ± 1.6 nA and 12.0 ± 0.2 nA, respectively). The discrimination error rate based on *I*_p_ was 4.7 ± 1.0%; thus, the accuracy was clearly improved compared to the results obtained by the microscopic examination in 0.5 × PBS (Table 1). The difference in the mean *I*_p_ for cancer cells and leukocytes was apparently larger than that of the difference obtained by microscopic examination (Table 2, Figure 2f). The decrease in current, which is denoted by *I*_p_, provides information of not only the particle size/volume, but also the deformability [1,6,7]. In addition to the difference in size between the two cell types, the difference in deformation must be significantly considered. CelSee Diagnostics uses both size exclusion and deformability to capture CTCs. However, a huge number of leukocytes are contaminated as a background in the captured cell population; immunocytochemistry, as well as nucleic acid fluorescence in situ hybridization, are employed for the identification and/or characterization of CTCs [15,30]. Although CTCs cannot be captured by RPM, an accurate discrimination and detection of cancer cells could be obtained by measuring the decrease in current, denoted by *I*_p_. The absolute value of the zeta potential decreases in a solution containing a large number of ions because the surface charge is strongly screened by the electrolyte ions [27]. As expected, the zeta potential of the cancer cells and leukocytes was enhanced to −13.5 ± 0.3 mV and −14.6 ± 0.1 mV, respectively, compared to those in 1.0 × PBS. Thereafter, the *t*_d_ parameter was examined (Figure 3f). The mean value and SD of *t*_d_ for the cancer cells and leukocytes were 136.4 ± 3.8 ms and 40.7 ± 1.2 ms, respectively (Table 2). The discrimination error rate in terms of the detection boundary for *t*_d_ (DB*_t_*_d_) was 6.7 ± 0.6% (Figure 3f, Table 1). The mean value and SD of *t*_d_ for the leukocytes were apparently lower than that in 1.0 × PBS. This may be attributed to a more accurate measurement of the surface charge of leukocytes through an increase in the electrophoretic force in a solution with a low electrolyte concentration. By performing discriminant analysis on the *I*_p_–*t*_d_ plane, in which the decision boundary is indicated by the broken line as DB*_I_*_p_–*_t_*_d_, we obtained a discrimination error rate of 4.5 ± 0.5% (Table 1). The accuracy of discrimination by *I*_p_–*t*_d_ indicated a significant improvement over *t*_d_ (McNemar test, *p* < 0.01). There is a tendency to improve the accuracy; however, there is no significant difference between *I*_p_–*t*_d_ and *I*_p_. The surface charge of the cells is considered to be less involved in cell discrimination compared to cell size and deformability in a solution with a low electrolyte concentration. All *I*_p_, *t*_d_, and *I*_p_–*t*_d_ analyses showed a more accurate cell discrimination in 0.5 × PBS compared to that in 1.0 × PBS, demonstrating the potential of RPM for an accurate cell discrimination in a solution with a low electrolyte concentration.

We also examined the applicability of RPM to other cell measurements. We showed the *I*_p_–*t*_d_ scatter plot by RPM analysis in 0.5 × PBS with a mixture of cancer cells and leukocytes in Figure S1. The results of measuring leukocytes and cancer cells separately and the results of measuring leukocytes and cancer cells in a mixed manner showed quite similar cell distribution results. Furthermore, we showed the *I*_p_–*t*_d_ scatter plot by RPM analysis of KATO-III and leukocytes in 0.5 × PBS (Figure S2). Similar cell distribution on *I*_p_–*t*_d_ scatter plot to that of NCI-1650 and leukocytes (Figure 3f) was observed, and accurate discrimination by *I*_p_–*t*_d_ analysis can be performed. These results show high applicability in other cell measurements.

## 4. Conclusions

In this study, the size of cancer cells was shown to be significantly larger than that of leukocytes in both the given electrolyte solutions. These results correspond well with those reported in previous studies [31]. The advantages of size-based CTC separation methods from blood include the fact that they are simple, fast, and inexpensive compared to the biological enrichment methods that are based on immunoreactions, including the CellSearch System, which is the only US Food and Drug Administration-approved CTC diagnosis system. However, the overlap in the size of different types of cells may cause a low accuracy. In this study, we demonstrated the potential of a microfluidic cell sensing chip, which is based on the RPM, for an accurate discrimination of cancer cells from leukocytes by measuring multiple parameters such as cell size, deformability, and surface charge in a solution with a low electrolyte concentration.

## Figures and Tables

**Table 1:** Discrimination of cancer cells from leukocytes in terms of cell size using microscopic examination, and that on *I*_p_, *t*_d_, and *I*_p_–*t*_d_ acquired by RPM.

**Discrimination Based on the Cell Size by a Microscopic Examination**						
**Actual Classification**	**Predicted Classification**					
	**1.0 × PBS**			**0.5 × PBS**		
	**Cancer Cells**	**Leukocytes**	**ER (%)**	**Cancer Cells**	**Leukocytes**	**ER (%)**
Cancer cells	71 (TP)	29 (FN)	17.5	90 (TP)	10 (FN)	8.5
Leukocytes	6 (FP)	94 (TN)		7 (FP)	93 (TN)	
Confidence interval of error rate (ER)	95% CI, 12.5–23.5			95% CI, 5.0–13.3		
**Discrimination Based on *I*_p_, or *t*_d_ Acquired by RPM**						
**Actual Classification**	**Predicted Classification by *I*_p_**					
	**1.0 × PBS**			**0.5 × PBS**		
	**Cancer Cells**	**Leukocytes**	**ER (%)**	**Cancer Cells**	**Leukocytes**	**ER (%)**
Cancer cells Leukocytes	163 34	87 216	24.2	237 12	13 238	5.0
Cancer cells Leukocytes	140 23	110 227	26.6	230 7	20 243	5.4
Cancer cells Leukocytes	122 27	128 223	31.0	238 7	12 243	3.8
Discrimination ER (mean ± SD %, *n* = 3)	27.3 ± 3.5			4.7 ± 0.8 **		
**Actual Classification**	**Predicted Classification by *t*_d_**					
Cancer cells Leukocytes	109 86	141 164	45.4	231 11	19 239	6.0
Cancer cells Leukocytes	96 70	154 180	44.8	224 9	26 241	7.0
Cancer cells Leukocytes	184 160	66 90	45.2	230 15	20 235	7.0
Discrimination ER	45.1 ± 0.3			6.7 ± 0.6 **		
**Discrimination Based on *I*_p_–*t*_d_ Acquired by RPM**						
**Actual Classification**	**Predicted Classification by *I*_p_–*t*_d_**					
	**1.0 × PBS**			**0.5 × PBS**		
	**Cancer Cells**	**Leukocytes**	**ER (%)**	**Cancer Cells**	**Leukocytes**	**ER (%)**
Cancer cells Leukocytes	202 23	48 227	14.2	238 11	12 239	4.6
Cancer cells Leukocytes	172 23	78 227	20.2	234 9	16 241	5.0
Cancer cells Leukocytes	161 20	89 230	21.8	240 10	10 240	4.0
Discrimination ER	18.7 ± 4.0			4.5 ± 0.5 **		

TP: true positive, FN: false negative, FP: false positive, TN: true negative. The CI for the discrimination error rate was evaluated using the Clopper–Pearson method. Significant differences were found in the results under 1.0 × PBS and 0.5 × PBS (** *p* < 0.01).

**Table 2:** Mean of *I*_p_ and *t*_d_ of each cell by RPM analysis.

	*I*_p_ (nA)		*t*_d_ (ms)	
	Cancer Cell	Leukocytes	Cancer Cell	Leukocytes
1.0 × PBS	58.7 ± 1.9	26.3 ± 1.6**	145.2 ± 1.9	150.0 ± 1.4 *
0.5 × PBS	50.8 ± 1.6	12.0 ± 0.2**	136.4 ± 3.8	40.7 ± 1.2 **

Data are expressed as the mean ± SD for three different experiments. Significantly different from each cell (* *p* < 0.05, ** *p* < 0.01).

## Data Availability

Not applicable.

## Supplementary Materials

The following are available online at https://www.mdpi.com/2079-6374/11/3/78/s1: Figure S1: *I*_p_–*t*_d_ scatter plot by RPM analysis in 0.5 × PBS with a mixture of cancer cells and leukocytes. Figure S2: RPM analysis of other cancer cells in 0.5 × PBS. (a) The microscopic images of KATO-III.

## References

[1] Song Y.X., Zhang J.Y., Li D.Q. (2017). Microfluidic and nanofluidic resistive pulse sensing: A review. Micromachines.

[2] Tsutsui M., Yoshida T., Yokota K., Yasaki H., Yasui T., Arima A., Tonomura W., Nagashima K., Yanagida T., Kaji N. (2017). Discriminating single-bacterial shape using low-aspect-ratio pores. Sci. Rep..

[3] Yusko E.C., Bruhn B.R., Eggenberger O.M., Houghtaling J., Rollings R.C., Walsh N.C., Nandivada S., Pindrus M., Hall A.R., Sept D. (2017). Real-time shape approximation and fingerprinting of single proteins using a nanopore. Nat. Nanotechnol..

[4] Ryuzaki S., Tsutsui M., He Y., Yokota K., Arima A., Morokawa T., Taniguchi M., Kawai T. (2017). Rapid structural analysis of nanomaterials in aqueous solutions. Nanotechnology.

[5] Arjmandi N., Van Roy W., Lagae L., Borghs G. (2012). Measuring the electric charge and zeta potential of nanometer-sized objects using pyramidal-shaped nanopores. Anal. Chem..

[6] Zheng Y., Nguyen J., Wang C., Sun Y. (2013). Electrical measurement of red blood cell deformability on a microfluidic device. Lab Chip.

[7] Darvish A., Goyal G., Aneja R., Sundaram R.V.K., Lee K., Ahn C.W., Kim K.-B., Vlahovska P.M., Kim M.J. (2016). Nanoparticle mechanics: Deformation detection via nanopore resistive pulse sensing. Nanoscale.

[8] Luo L., German S.R., Lan W.-J., Holden D.A., Mega T.L., White H.S. (2014). Resistive-pulse analysis of nanoparticles. Annu. Rev. Anal. Chem..

[9] Wanunu M. (2012). Nanopores: A journey towards DNA sequencing. Phys. Life Rev..

[10] Howorka S., Siwy Z. (2009). Nanopore analytics: Sensing of single molecules. Chem. Soc. Rev..

[11] Xu Y., Xie X., Duan Y., Wang L., Cheng Z., Cheng J. (2016). A review of impedance measurements of whole cells. Biosens. Bioelectron..

[12] Fidler I.J. (2003). Timeline—The pathogenesis of cancer metastasis: The ‘seed and soil’ hypothesis revisited. Nat. Rev. Cancer.

[13] Sieuwerts A.M., Kraan J., Bolt J., van der Spoel P., Elistrodt F., Schutte M., Martens J.W.M., Gratama J.-W., Sleijfer S., Foekens J.A. (2009). Anti-epithelial cell adhesion molecule antibodies and the detection of circulating normal-like breast tumor cells. J. Natl. Cancer Inst..

[14] Mostert B., Kraan J., Bolt-de Vries J., van der Spoel P., Sieuwerts A.M., Schutte M., Timmermans A.M., Foekens R., Martens J.W.M., Gratama J.-W. (2011). Detection of circulating tumor cells in breast cancer may improve through enrichment with anti-CD146. Breast Cancer Res. Treat..

[15] van der Toom E.E., Verdone J.E., Gorin M.A., Pienta K.J. (2016). Technical challenges in the isolation and analysis of circulating tumor cells. Oncotarget.

[16] Tsutsui M., He Y., Furuhashi M., Rahong S., Taniguchi M., Kawai T. (2012). Transverse electric field dragging of DNA in a nanochannel. Sci. Rep..

[17] Sibarani J., Takai M., Ishihara K. (2007). Surface modification on microfluidic devices with 2-methacryloyloxyethyl phosphorylcholine polymers for reducing unfavorable protein adsorption. Colloids Surf. B. Biointerfaces.

[18] Fukazawa K., Ishihara K. (2012). Simple surface treatment using amphiphilic phospholipid polymers to obtain wetting and lubricity on polydimethylsiloxane-based substrates. Colloids Surf B Biointerfaces.

[19] Pischel D., Buchbinder J.H., Sundmacher K., Lavrik I.N., Flassig R.J. (2018). A guide to automated apoptosis detection: How to make sense of imaging flow cytometry data. PLoS ONE.

[20] Fraley C., Raftery A.E. (2002). Model-based clustering, discriminant analysis, and density estimation. J. Am. Stat. Assoc..

[21] Wernecke K.-D., D’Agostino R.B., Sullivan L., Massaro J. (2007). Discriminant Analysis. Wiley Encyclopedia of Clinical Trials.

[22] Ballabio D., Todeschini R., Sun D.-W. (2009). Multivariate Classification for Quantitative Analysis. Infrared Spectroscopy for Food Quality Analysis and Control.

[23] Fagerland M.W., Lydersen S., Laake P. (2014). Recommended tests and confidence intervals for paired binomial proportions. Stat. Med..

[24] Smeets R.M., Keyser U.F., Dekker N.H., Dekker C. (2008). Noise in solid-state nanopores. Proc. Natl. Acad. Sci. USA.

[25] Bondar O.V., Saifullina D.V., Shakhmaeva I.I., Mavlyutova I.I., Abdullin T.I. (2012). Monitoring of the zeta potential of human cells upon reduction in their viability and interaction with polymers. Acta Nat..

[26] Dobrzynska I., Skrzydlewska E., Figaszewski Z.A. (2013). Changes in electric properties of human breast cancer cells. J. Membr. Biol..

[27] Schoch R.B., Han J.Y., Renaud P. (2008). Transport phenomena in nanofluidics. Rev. Mod. Phys..

[28] de Wit S., van Dalum G., Lenferink A.T.M., Tibbe A.G.J., Hiltermann T.J.N., Groen H.J.M., van Rijn C.J.M., Terstappen L. (2015). The detection of EpCAM^+^ and EpCAM^-^ circulating tumor cells. Sci. Rep..

[29] Rostami P., Kashaninejad N., Moshksayan K., Saidi M.S., Firoozabadi B., Nguyen N.T. (2019). Novel approaches in cancer management with circulating tumor cell clusters. J. Sci. Adv. Mater. Dev..

[30] Gogoi P., Sepehri S., Zhou Y., Gorin M.A., Paolillo C., Capoluongo E., Gleason K., Payne A., Boniface B., Cristofanilli M. (2016). Development of an Automated and Sensitive Microfluidic Device for Capturing and Characterizing Circulating Tumor Cells (CTCs) from Clinical Blood Samples. PLoS ONE.

[31] Austin R.G., Huang T.J., Wu M., Armstrong A.J., Zhang T. (2018). Clinical utility of non-EpCAM based circulating tumor cell assays. Adv. Drug Deliv. Rev..

