# Supplementary material for: Effect of Electrolyte Concentration on Cell Sensing by Measuring Ionic Current Waveform through Micropores
Source: Biosensors (Basel). 2021 Mar 12;11(3):78. doi: 10.3390/bios11030078 (PMC7998150; doi:10.3390/bios11030078)
Supplement: Supplementary file 1 [file biosensors-11-00078-s001.pdf]

biosensors

MDPI

Supproting information

Effect of electrolyte concentration on cell sensing by measuring ionic current waveform through micropore

Kazumichi Yokota [1], Muneaki Hashimoto [1], Kazuaki Kajimoto [1], Masato Tanaka [1], Sanae Murayama [2], Makusu Tsutsui [2], Yoshihiro Nakajima [1], Masateru Taniguchi [2] and Masatoshi Kataoka [1,*]

[1] National Institute of Advanced Industrial Science and Technology, Takamatsu, Kagawa 761-0395, Japan; kazumichi-yokota@aist.go.jp (K.Y.); muneaki-hashimoto@aist.go.jp (M.H.); k-kajimoto@aist.go.jp (K.K.); mst-tanaka@aist.go.jp (M.T.); y-nakajima@aist.go.jp (Y.N.)

[2] The Institute of Scientific and Industrial Research, Osaka University, 8-1 Mihogaoka, Ibaraki, Osaka 567-0047, Japan; murayama@sanken.osaka-u.ac.jp (S.M.); tsutsui@sanken.osaka-u.ac.jp (M.T.); taniguti@sanken.osaka-u.ac.jp (M.T.)

* Correspondence: m-kataoka@aist.go.jp; Tel.: +81-87-869-3576

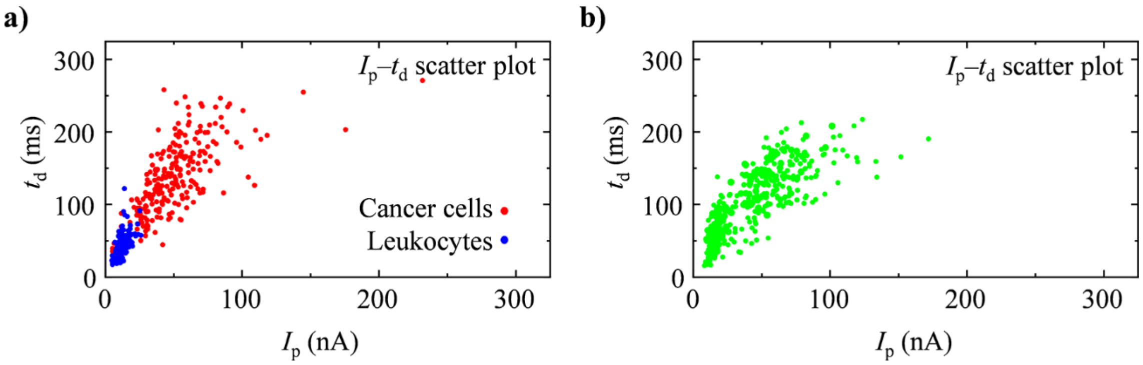

Figure S1. I_p-t_d scatter plot by RPM analysis in 0.5 × PBS with a mixture of cancer cells and leukocytes. (**a**) The results of measuring leukocytes and cancer cells separately. (**b**) The results of measuring leukocytes and cancer cells in a mixed manner. Similar cell distribution could be observed when cells were measured separately.

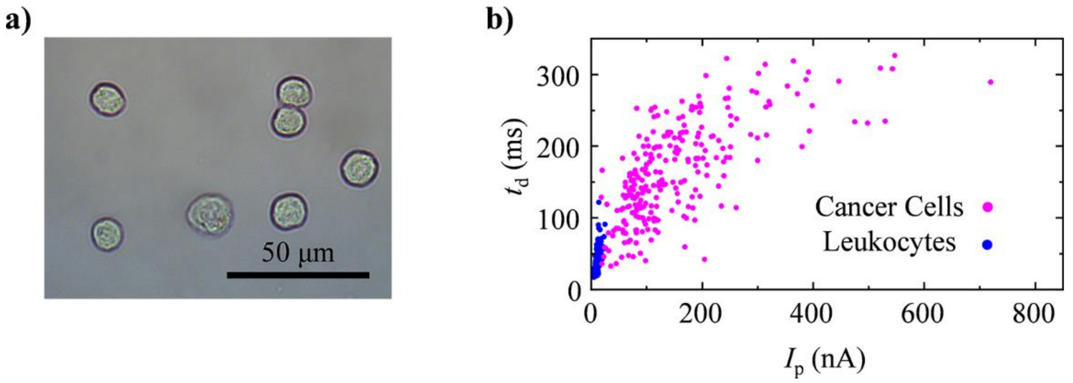

Figure S2. RPM analysis of other cancer cells in 0.5 × PBS. (**a**) The microscopic images of KATO-III. To determine the size of KATO-III cells, 100 cells were examined using an optical microscope. The average value and SD for cell diameters of KATO-III cells were 15.8 ± 4.7 µm, which were larger than NCI-H1650 cells. (**b**) Scatter plot of I_p-t_d for leukocytes and KATO-III cells. As a whole, the similar cell distribution as in the case of NCI-H1650 cells is observed, and it is possible to accurately discrimination it from leukocytes. The I_p value tends to be clearly enhanced, reflecting the size of the cell size.
